# Evolution of phenotypic plasticity owing to migration

**DOI:** 10.1093/evlett/qraf040

**Published:** 2025-11-08

**Authors:** Davorka Gulisija, Mitchell Newberry

**Affiliations:** Department of Biology, University of New Mexico, Albuquerque, United States; Department of Computer Science, University of New Mexico, Albuquerque, United States; Department of Biology, University of New Mexico, Albuquerque, United States

**Keywords:** phenotypic plasticity, population structure, migration

## Abstract

Phenotypic plasticity enables organisms to produce better-suited phenotypes when the environment changes, improving fitness under adverse conditions. Yet responding to environmental cues may provide little use in a constant environment, where organisms already express optimal phenotypes. The forces that sustain plasticity and account for its widespread presence, thus, remain unclear, as plasticity must remain advantageous to persist. Although typically associated with changing environments, maintenance of plasticity requires generational turnover such that parents and offspring regularly encounter different conditions. Here, we demonstrate that even a low number of migrants between locally adapted populations, in constant environments, can promote the emergence and persistence of phenotypic plasticity even when plasticity is costly and never associated with the fittest genotype, independent of its genetic architecture. We support this conclusion by exploring the parameter space of a two-locus, two-deme model using stochastic simulations and analytical approximations. We derive analytical conditions under which plasticity is adaptively maintained as a function of selection strength, migration, and fitness trade-offs. These findings reveal new potential evolutionary origins of plasticity and offer insight into how maladaptive traits can invade adapted populations in stable environments.

## Teaser

Phenotypes can change without alterations in the underlying genotype—a phenomenon known as phenotypic plasticity—the evolution of which is typically triggered by adverse environmental conditions. Consequently, when we observe adapted populations in stable habitats, we do not expect phenotypic plasticity to be maintained by selection. Here, we show that migration introducing maladapted phenotypes, which may be rapidly removed by selection, can enable plasticity to invade adapted populations in stable environments. This holds even when plasticity is costly, not associated with the fittest genotype, and regardless of its genetic architecture. Migration contributes to the long-term maintenance of plasticity and may explain seemingly maladaptive plastic responses in stable habitats.

## Introduction

Phenotypic plasticity is the ability of a genotype to produce different phenotypes in response to environmental cues (Pigliucci et al., 2006; Scheiner, 1993), allowing populations to respond rapidly to environmental change. With the increasing impact of global climate change (Martinich & Crimmins, 2019; Rahmstorf & Coumou, 2012), habitat destruction ( Hanski, 2011), and invasions (Seebens et al., 2013; Sardain et al., 2019), understanding mechanisms that promote phenotypic plasticity (hereafter plasticity) is crucial for predicting future biodiversity. Here, we propose a new mechanism in which plasticity emerges and is maintained as a result of migration between locally adapted populations.

Populations differ in degree of plasticity (de la Matta et al., 2022; Hattich et al., 2017; Porlier et al., 2012; Sommer et al., 2017), reflecting underlying genetic differences shaped by distinct evolutionary processes (Scheiner, 1993). The rapid emergence of plasticity in some populations suggests that plasticity-conferring genes may already be present at the time of environmental change (Promy et al., 2023; Scoville & Pfrender, 2010). However, plasticity is favored by selection only if it provides a fitness benefit, which raises the question of what evolutionary mechanisms might maintain plasticity in the presence of non-plastic, locally adapted phenotypes (Hollander & Butlin, 2010), even before environments change (Wright et al., 2016).

Moreover, in the presence of non-plastic optimal phenotypes, plasticity may become maladaptive. This is because plasticity can incur fitness costs (Ancel, 2000; DeWitt et al., 1998; Schlichting & Pigliucci, 1998; Lande, 2009), arising from, e.g., maintenance of sensory machinery (Kussell & Leibler, 2005), regulatory complexity (Scheiner, 1993), or development and maintenance of systems involved in osmo- or thermo-regulation (Krebs & Feder, 1997). The prevalence of such costs is difficult to assess since we tend to observe plasticity when it is adaptive, where the benefits outweigh the costs. While the prevalence of plasticity costs is still debated (Murren et al., 2015; Little & Seebacher, 2025), the observation that populations in stable habitats exhibit lower levels of plasticity than those in perturbed environments (Lande, 2009; de la Mata et al., 2022) indirectly suggests that plasticity incurs some cost. At best, in the absence of cost, plasticity alleles would be neutral after adaptation to a non-plastic phenotype and drift in frequency. That plasticity remains widespread is therefore surprising, given the potential costs of maintaining plastic responses. It remains unclear what mechanisms account for the widespread maintenance of plasticity in nature.

For plasticity to retain its selective advantage, populations must experience adverse conditions regularly. One well-established mechanism promoting plasticity is temporally varying selection (Gulisija et al., 2016), such as populations that experience seasonal shifts over multiple generations (Bergland et al., 2014). In these cases, a non-plastic adaptation may take longer than the timescale of environmental shifts (Gulisija et al., 2016). It is, however, important to distinguish temporally varying selection from within-lifetime environmental variation, where individuals experience changing conditions across their lifespan. For organisms with longer generation times, temporally varying selection may not be a sufficient explanation for the persistence of plasticity, as longer lifespans also buffer against environmental variation (Gamelon et al., 2014; Le Coeur et al., 2022). Instead, plasticity in these organisms may arise, at least temporarily, de novo through beneficial mutations during colonization of new adverse habitats (Kapun et al., 2019). Finally, the degree of polygenic plasticity may increase, even in locally adapted populations, if plastic immigrants enter the population (Chevin & Lande, 2011; Sultan & Spencer, 2002), though this assumes standing genetic variation for plasticity, bringing us back to the original question of how such variation is maintained in the source population.

What is underexplored is whether plasticity could evolve *de novo* in locally adapted populations due to migration. Here, plasticity seemingly offers no benefit to most individuals in the population. Plasticity may be valuable to maladaptive migrants, but plasticity alleles can recombine onto locally-adapted genetic backgrounds where they no longer provide a benefit or may even be selected against due to the cost of plasticity. Thus, the evolutionary fate of plasticity-related alleles in locally adapted populations exchanging migrants remains unclear. The emergence and maintenance of plasticity driven by migration under conditions that favor local adaptation would broaden the theoretical conditions for its evolution and explain its widespread presence in nature.

We therefore study the emergence of plasticity owing to the exchange of migrants between populations adapted to local conditions. We propose that although the cost or limited benefit of plasticity may prevent them from being associated with the fittest genotypes, plastic alleles may, on average, surpass non-plastic alleles in fitness within a mixed population that includes maladapted immigrants. This case may explain the emergence of plasticity in many populations, irrespective of their generation times, and doesn’t require that the entire population experiences habitat shift.

**Figure 1.**
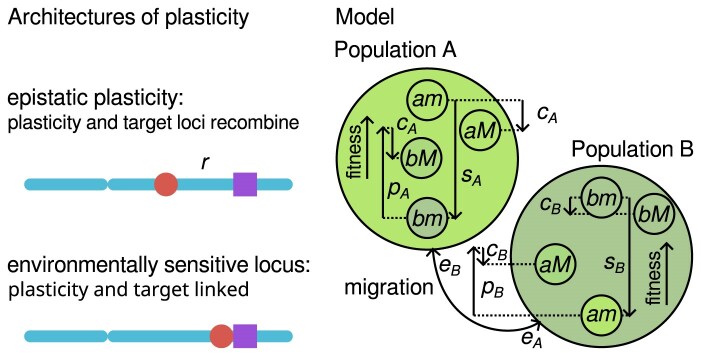
The architecture and the effects of phenotypic plasticity. Left: Two empirically supported models of the genetic basis of plastic control: epistatic plasticity, where the plasticity modifier locus (such as epigenetic modifier, purple) and target locus (non-plastic phenotype gene, red) recombine and interact, and environmentally sensitive locus, where the target phenotype and its plasticity are properties of the same locus. Right: Model schematic showing how the plasticity locus and target locus jointly determine individual fitness in two locally adapted populations, A and B, that exchange migrants. Maladapted immigrants, *a* in B and *b* in A, gain a fitness advantage *p_i_*–*c_i_* from association with the plasticity allele, *M*, compared to the non-plastic allele, *m*, whereas adapted residents, *a* in A and *b* in B, incur a fitness cost, *c_i_*, for carrying *M*.

To study the evolution of plasticity, we use a modifier allele approach (Figure 1, left; Carja & Feldman, 2012; Draghi & Whitlock, 2012; Gulisija et al., 2016), which enables us to distinguish between trait plasticity, assigned to a plasticity modifier locus, and the expected non-plastic trait value, assigned to a target locus. Models where the modifier and target loci recombine correspond to a genetic architecture known as epistatic plasticity (Carja & Feldman, 2012; Draghi & Whitlock, 2012; Gulisija et al., 2016). Conversely, if we assume no recombination between these loci, the model corresponds to environmentally sensitive loci (Scheiner, 1993; Via & Lande, 1985). Under the former architecture, plasticity-modifier loci, such as major effect loci, interact with or regulate expression at other loci to influence phenotypes in an environmentally dependent manner. This architecture of phenotypic plasticity has been supported by studies in several model species (Bergland et al., 2008; Bhatia et al., 2014; Leips & Mackay, 2000; Queitsch et al., 2002; Rutherford & Lindquist, 1998; Stratton, 1998; Tetard-Jones et al., 2011). On the other hand, the environmentally sensitive architecture involves loci that are directly regulated by the environment, such as the *lac* operon in bacteria (Jacob & Monod, 1961), or cases where environmental cues induce epigenetic modifications near coding sequences (Beldade et al., 2011; Herman & Sultan, 2016). By modeling recombination or complete linkage between a plasticity modifier locus and a target locus, the modifier framework enables investigation of the evolution of plasticity under both empirically supported genetic architectures.

We examine the effects of varying strengths of opposing selection pressures between two populations, migration rates, and the benefits and costs of plasticity under favorable and adverse conditions. We also assess the robustness of these effects under perturbations to model assumptions. We derive analytical conditions for the maintenance of plasticity in terms of the strength of selection, migration, and fitness costs and benefits of plasticity. Our results reveal a range of conditions under which migration drives the emergence and maintenance of plasticity in finite populations, even when plasticity is costly or yields suboptimal benefit.

## Model and methods

We develop a two-locus, two-deme population genetic model in which alleles *a* and *b* at one locus code for a non-plastic adaptive phenotype (structural gene) in demes A and B, while alleles *m* and *M* at a second locus (plasticity modifier locus) determine whether there is a plastic environmental response. Rather than modeling individual phenotypes or reaction norms, we directly specify the fitness effects of each haplotype in each population and model the evolutionary dynamics at the two loci independently of the rest of the genome (Ewens, 2004; Gillespie, 2004). Furthermore, by specifying the fitness costs and benefits of plasticity, we model the full range of plausible mechanisms, such as the energetic or other costs associated with maintaining the physiological machinery underlying plasticity (Krebs & Feder, 1997). For example, we account for maladaptive plasticity in favorable conditions by imposing a fitness cost on otherwise well-adapted carriers (Figure 1, right). Likewise, tuning recombination rates (*r*) between the two loci, our model accounts for both of the empirically supported models of plastic control: epistatic plasticity (such as modeled in Carja & Feldman, 2012; Draghi & Whitlock, 2012; and Gulisija et al., 2016), as well as environmentally sensitive locus models of plasticity (Scheiner, 1993; Via & Lande, 1985), when the plasticity and non-plastic expression are coupled (Figure 1, left).

We investigate the dynamics of frequencies of the four possible haplotypes in each population *x_amj_, x_aMj_, x_bmj_*, and *x_bMj_*, where *j* = A or B, evolving forward in time under spatially heterogeneous selection, recombination between the two loci, genetic drift, and migration between the two Wright-Fisher populations (Figure 1, right). We initiate simulations with two locally adapted populations A and B, each monomorphic for the *a* and *b* alleles, respectively, and both initially fixed for the non-plasticity allele (*m*). The populations continuously exchange migrants: the migration rate *e_A_* denotes individuals from population A that migrate to population B, while *e_B_* represents migration in the opposite direction. Once the migration-selection equilibrium is achieved, we examine whether a plasticity modifier allele invades the metapopulation. We allow a single copy of a novel plasticity mutant allele (*M*) to arise at the modifier locus in a random individual in one of the two populations. A single mutant introduction allows us to examine the dynamics at the plasticity modifier locus under the regime of the rare beneficial mutation although we later relax this assumption by modeling recurrent mutation.

Selection acts to decrease the fitness of the immigrants compared to the residents, with *s_j_* denoting a selection coefficient in population *j*. Plasticity buffers this selection on immigrants carrying the plastic allele *M*, which we represent as a fitness benefit *p_j_* in the hostile deme, but plasticity may incur a fitness cost *c_j_* to its carriers (Figure 1, right). If paired with the locally adaptive target allele, plasticity offers no benefit, just cost. These very general assumptions on selection result in the following fitness effects, *w_ij_*, for each haplotype *i* (*am, aM, bm, bM*) in each population *j* (A or B):

**Table:** 

	Population A	Population B
Resident	${{{\mathrm{w}}}_{am{\mathrm{A}}}} = 1$	${{{\mathrm{w}}}_{bm{\mathrm{B}}}} = 1$
Plastic resident	${{{\mathrm{w}}}_{aM{\mathrm{A}}}} = 1 - {{c}_{\mathrm{A}}}$	${{{\mathrm{w}}}_{bM{\mathrm{B}}}} = 1 - {{c}_{\mathrm{B}}}$
Immigrant	${{{\mathrm{w}}}_{bm{\mathrm{A}}}} = 1 - {{s}_{\mathrm{A}}}$	${{{\mathrm{w}}}_{am{\mathrm{B}}}} = 1 - {{s}_{\mathrm{B}}}$
Plastic immigrant	${{{\mathrm{w}}}_{bM{\mathrm{A}}}} = 1 - {{s}_{\mathrm{A}}} + {{p}_{\mathrm{A}}} - {{c}_{\mathrm{A}}}$	${{{\mathrm{w}}}_{aM{\mathrm{B}}}} = 1 - {{s}_{\mathrm{B}}} + {{p}_{\mathrm{B}}} - {{c}_{\mathrm{B}}}$

With average fitness in population *j* at time *t* given by ${{{\mathrm{\bar{w}}}}_{j,\ t}} = {{{\mathrm{x}}}_{amj{\mathrm{,\ }}t}}{{{\mathrm{w}}}_{amj}} + {{{\mathrm{x}}}_{aMj{\mathrm{,\ }}t}}{{{\mathrm{w}}}_{aMj}} + {{{\mathrm{x}}}_{bmj{\mathrm{,\ }}t}}{{{\mathrm{w}}}_{bmj}} + {{{\mathrm{x}}}_{bMj{\mathrm{,\ }}t}}{{{\mathrm{w}}}_{bMj\ }}$, the expected post-selection haplotype frequencies in generation *t* become ${\mathrm{x}}{{{\mathrm{^{\prime}}}}_{amj,\ t}} = {{{\mathrm{x}}}_{amj,\ t}}\frac{{{{{\mathrm{w}}}_{amj}}}}{{{{{{\mathrm{\bar{w}}}}}_{j,\ t}}}},{\mathrm{\ \ x}}_{aMj,\ t}^{\mathrm{^{\prime}}} = {{{\mathrm{x}}}_{aMj,\ t}}\frac{{{{{\mathrm{w}}}_{aMj}}}}{{{{{{\mathrm{\bar{w}}}}}_{j,\ t}}}},{\mathrm{\ x}}_{bmj,\ t}^{\mathrm{^{\prime}}} = {{{\mathrm{x}}}_{bmj,\ t}}\frac{{{{{\mathrm{w}}}_{bmj}}}}{{{{{{\mathrm{\bar{w}}}}}_{j,\ t}}}},{\mathrm{\ and\ x}}_{bMj,\ t}^{\mathrm{^{\prime}}} = {{{\mathrm{x}}}_{bMj,\ t}}\frac{{{{{\mathrm{w}}}_{bMj}}}}{{{{{{\mathrm{\bar{w}}}}}_{j,\ t}}}}$.

We constrain the parameter space to *s_j_* ≥ *p_j_* ≥ *c_j_* ≥ 0. These constraints place the fitness of plastic haplotypes within the range spanning optimally adapted non-plastic residents (fittest) and maladapted, non-plastic invaders (least fit). For example, in the absence of cost with *s_j_ = p_j_*, plastic invaders and non-plastic residents have equal fitness. Otherwise, plastic invaders are less fit than residents, but more fit than non-plastic invaders. With any non-zero cost to plasticity or *s_j_* > *p_j_*, non-plastic residents are always the fittest haplotype.

Note that the above framework assigns a single effect value to the benefit and cost of plasticity within a given generation. We do not assume whether this results from permanent, lifelong developmental plasticity (Scheiner, 1993) or the marginal value of plastic responses to within-lifetime changes or life-stage transitions. Moreover, we do not consider scenarios in which the plastic phenotype is beneficial under every condition, since such forms of plasticity would always evolve. In contrast, our focus is on plastic responses that may evolve in some populations in response to fitness perturbations.

Following selection, the expected haplotype frequencies ${\mathrm{x}}_{amj{\mathrm{,}}t}^{\mathrm{^{\prime}}},{\mathrm{\ \ x}}_{aMj{\mathrm{,}}t}^{\mathrm{^{\prime}}},{\mathrm{\ x}}_{bmj{\mathrm{,}}t}^{\mathrm{^{\prime}}},{\mathrm{\ and\ x}}_{bMj,\ t}^{\mathrm{^{\prime}}}$ at time *t* are altered by recombination in each population. With the coefficient of linkage disequilibrium ${{D}_{j,t}} = {\mathrm{x}}_{amj{\mathrm{,}}t}^{\mathrm{^{\prime}}}{\mathrm{x}}_{bMj{\mathrm{,}}t}^{\mathrm{^{\prime}}} - {\mathrm{\ x}}_{aMj{\mathrm{,}}t}^{\mathrm{^{\prime}}}{\mathrm{x}}_{bmj{\mathrm{,}}t}^{\mathrm{^{\prime}}}$, the expected post-recombination frequencies of four haplotypes are


\begin{eqnarray*}
x_{amj,t}^{{\mathrm{^{\prime\prime}}}} = x_{amj{\mathrm{,}}t}^{\mathrm{^{\prime}}} - {{D}_{j,t}}r,
\end{eqnarray*}

\begin{eqnarray*}
x_{aMj{\mathrm{,}}t}^{{\mathrm{^{\prime\prime}}}} = x_{aMj{\mathrm{,}}t}^{\mathrm{^{\prime}}} + {{D}_{j,t}}r,
\end{eqnarray*}

\begin{eqnarray*}
x_{bmj{\mathrm{,}}t}^{{\mathrm{^{\prime\prime}}}} = x_{bmj{\mathrm{,}}t}^{\mathrm{^{\prime}}} + {{D}_{j,t}}r,
\end{eqnarray*}

and


\begin{eqnarray*}
x_{bMj{\mathrm{,}}t}^{{\mathrm{^{\prime\prime}}}} = x_{bMj{\mathrm{,}}t}^{\mathrm{^{\prime}}} - {{D}_{j,t}}r,
\end{eqnarray*}

where *r* is the recombination rate between the plasticity modifier and the target locus, which is equal in the two demes.

Finally, to account for genetic drift in finite populations, we sample *N_j_* individuals from a multinomial distribution for the next generation, proportional to the expected haplotype frequencies ${\mathrm{x}}_{amj{\mathrm{,}}t}^{{\mathrm{^{\prime\prime}}}},{\mathrm{\ \ x}}_{aMj{\mathrm{,}}t}^{{\mathrm{^{\prime\prime}}}},{\mathrm{\ x}}_{bmj{\mathrm{,}}t}^{{\mathrm{^{\prime\prime}}}},{\mathrm{\ and\ x}}_{bMj{\mathrm{,}}t}^{{\mathrm{^{\prime\prime}}}}$, generating the sampled frequencies in the subsequent generation ${{{\mathrm{x}}}_{amj{\mathrm{,\ }}t{\mathrm{ + 1}}}},{\mathrm{\ }}{{{\mathrm{x}}}_{aMj{\mathrm{,\ }}t{\mathrm{ + 1}}}},{\mathrm{\ }}{{{\mathrm{x}}}_{bmj{\mathrm{,\ }}t{\mathrm{ + 1}}}},{\mathrm{\ and\ }}{{{\mathrm{x}}}_{bMj{\mathrm{,\ }}t{\mathrm{ + 1}}}}$.

We track the frequency of the plasticity modifier allele and define adaptive plasticity when its fixation rate (single mutant introduction) or expected frequency (recurrent mutation) exceeds the neutral expectation. Our central assumption is that, for plasticity to be maintained by selection, it must confer a continuous adaptive advantage.

### Implementation and simulation details

Using stochastic forward-in-time Monte Carlo simulations, we simulate a wide range of evolutionary scenarios in haploid Wright-Fisher populations. We initiate each simulation run (at a discrete generation *t* = 0), assuming two locally adapted non-plastic populations at the onset of migration. We allow a burn-in period during which migration between the two populations occurs to achieve an equilibrium between selection and migration, defined as a change in allele frequency over 100 generations < 0.001, across simulation runs. Once the migration-selection equilibrium is achieved, we examine if a plasticity mutant allele (*M*) will invade either of the adapted populations while experiencing migration, selection, recombination, and genetic drift.

We study the evolution of plasticity when a single plasticity modifier allele enters one of two randomly chosen, locally adapted populations at migration–selection equilibrium. We use the fixation probability of the invading allele, in the absence of clonal interference, as a proxy for the adaptive effect of plasticity under a rare mutation regime. We relax this assumption by allowing for recurrent mutation, where each of the target and plasticity alleles mutates from locally adaptive to non-adaptive target alleles, and from plastic to non-plastic alleles, and vice versa, with probability $\mu$ in each generation. We examine evolutionary dynamics under no recombination between the target and the plasticity locus (environmentally sensitive loci), and under the closely linked, loosely linked, or unlinked epistatically interacting target and plasticity locus. We study a variety of selective regimes, benefits, and costs of plasticity (Table 1). We initially set ${{s}_{\mathrm{A}}} = {{s}_{\mathrm{B}}}$, but later relax this assumption to accommodate uneven magnitudes of selective pressures, benefit and cost of plasticity, as well as differences in migration rates between the two populations.

**Table 1.** List of major parameters and their ranges.

Parameter	Description and range
*N_j_*	size of population *j* (*j* = A or B), *N*_A_ = 10000 or 5000, *N*_B_ = 0.2, 0.5, or 1 × *N*_A_
*e_j_*	number of emigrants from population *j*, *e*_A_ = 1, 3, 5, 8, 10, 15, 20, 25, 50, 75,100, 200,300, 500,750, 1000,1500, 2000,2500, 3000,3500, 4000,4500, or 5000, *e*_B_ = ½, 1/3, or 1 × *e*_A_, per generation; *e*_A_ = *e*_B_ = 1, 5, or 10, periodically each 1, 2, 3, 4, 5, 10, 15, 20, 25, 50, or 100 generations corresponding to (number of migrants)/(interval between migration events) per generation.
*s_j_*	negative selection effect on the immigrant target allele in population *j*, *s*_A_ = 0.01, 0.03 or 0.05; *s*_B_ = 1/3, ½, or 1 × *s*_A_
*p_j_*	fitness benefit of plasticity (*M*) to an immigrant allele, *p_j_* = *s_j_, s_j_*/2, or 0.
*c_j_*	cost of plasticity to the resident genotype, *c_j_* = 0, *p_j_*/4, or *p_j_*/3.
*r*	recombination rate between the plasticity modifier and the target locus, *r* = 0.001, 0.1, or 0.5, representing epistatic plasticity, or *r* = 0 representing environmentally sensitive locus.
*μ*	recurrent mutation rate at the two loci per generation in each population, *μ* = 0.001/*N_j_*

To ensure convergence, we perform forward-in-time simulations (Gulisija & Newberry, 2025), with each run spanning 100(*N*_A_ *+ N*_B_) replicates or 20000 replicates in the case of recurrent mutation, where *N_j_* denotes the number of individuals in population *j*. Simulations continue until fixation or loss of the *M* allele following its introduction at a single locus, or for 100(*N*_A_ *+ N*_B_) generations post burn-in period to assure stability of the system in the face of recurrent mutation at both loci in both populations.

For single allele introduction simulation runs, we record the fixation probability of a newly introduced plasticity modifier allele at a metapopulation level, fixation and loss times, and heterozygosity for the plasticity locus, under both selection and the neutral control (drift, *s_j_* = 0). Under recurrent mutation, we record equilibrium frequencies of the plasticity allele at the metapopulation levels, under selection and neutrality.

## Results

The exchange of migrants between two locally adapted populations can drive the evolution of adaptive plasticity, with plasticity-conferring alleles (*M*) sweeping to fixation in both populations, even when plasticity is not associated with the fittest genotype. When plasticity incurs a fitness cost, higher migration rates are required. Moreover, the evolution of plasticity via migration is relatively robust to asymmetries in selection strength, plasticity benefits, migration rates, population sizes, the mode of plastic control, and recurrent mutation, allowing plasticity to persist stably over long evolutionary timescales. The results also suggest that adaptive plasticity hinges on reciprocal migration. Finally, we derive an analytical solution that frames the evolution of adaptive plasticity in terms of local adaptation strength, migration, and the fitness costs and benefits of plasticity.

### Migration promotes plasticity in locally adapted populations

**Figure 2.**
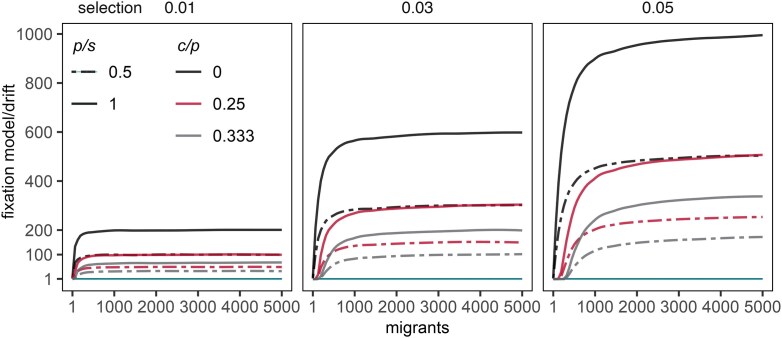
Emergence of plasticity owing to migration. Fixation probability of a plasticity modifier allele relative to that under drift (> 1 = adaptive plasticity, with horizontal green line depicting neutral expectation) as a function of the number of migrants between populations and selection coefficient (*s*_A_ = *s*_B_ = *s*), plasticity benefit (*p*_A_ = *p*_B_ = *p*), and the cost of plasticity (*c*_A_ = *c*_B_ = *c*). Simulations assumed that a single plasticity modifier allele is introduced to a monomorphic non-plastic meta-population and evolved forward-in-time until the allele is fixed or lost over 1000(*N*_A_ + *N*_B_) replicate runs, assuming *N*_A_ = *N*_B_ = 10,000 individuals and free recombination between the plasticity and target locus (epistatic plasticity). Figure S1 shows the effect with a focus on a low number of migrants.

Phenotypic plasticity can arise *de novo* in locally adapted populations, provided there is ongoing migration between them. We initially discuss the effect under single mutant introduction, which allows us to examine the fixation probability of a plastic allele *M* compared to the theoretical expectation under neutrality in the absence of clonal interference (Figure 2). In the absence of plasticity cost, the plasticity modifier allele fixes readily at the metapopulation level across the range of migration rates, at rates notably exceeding neutral expectations, indicating that plasticity is adaptive. Remarkably, the effect arises even when the benefit of plasticity provides only partial protection from negative selection and the resident non-plastic genotype is always the fittest (Figure 2, broken lines). With some cost to plasticity, the effect requires increasing migration, depending on selection strength (*s_j_*) and the relative benefits and costs (grey and red lines, Figure 2). The overall advantage of plasticity increases with the selection strength and the benefit of plasticity.

Investigating a lower number of migrants (Figure S1), we find that costly plasticity is maladaptive. The threshold rates at which plasticity becomes adaptive range from proportion of migrants of around 0.005*N_j_* with lower cost and milder selection (*c_j_/p_j_* = 1/4, *s_j_* = 0.01) to over 0.03*N_j_* with relatively higher cost and stronger selection (*c_j_/p_j_* = 1/3, *s_j_* = 0.05), indicating that the minimal number of migrants needed for adaptive plasticity quickly increases with fitness tradeoffs, but with little effect of the relative magnitude of the benefit. Beyond a critical migration rate, plasticity retains an adaptive effect, including complete panmixia (migrants = 0.5*N_j_*; Figure 1).

Recombination rates between the plasticity modifier and target locus have little effect on the adaptive status of plasticity (Figure S2). In general, while plasticity remains adaptive across a similar range of parameters as under free recombination (*r* = 0.5), tight linkage (*r* = 0.001) slightly reduces the fixation probability and increases the time to fixation of the plasticity modifier allele. We did not explore complete linkage (*r* = 0.0) under a single mutant introduction, as it creates an unrealistic case where alleles cannot spread across genetic backgrounds.

**Figure 3.**
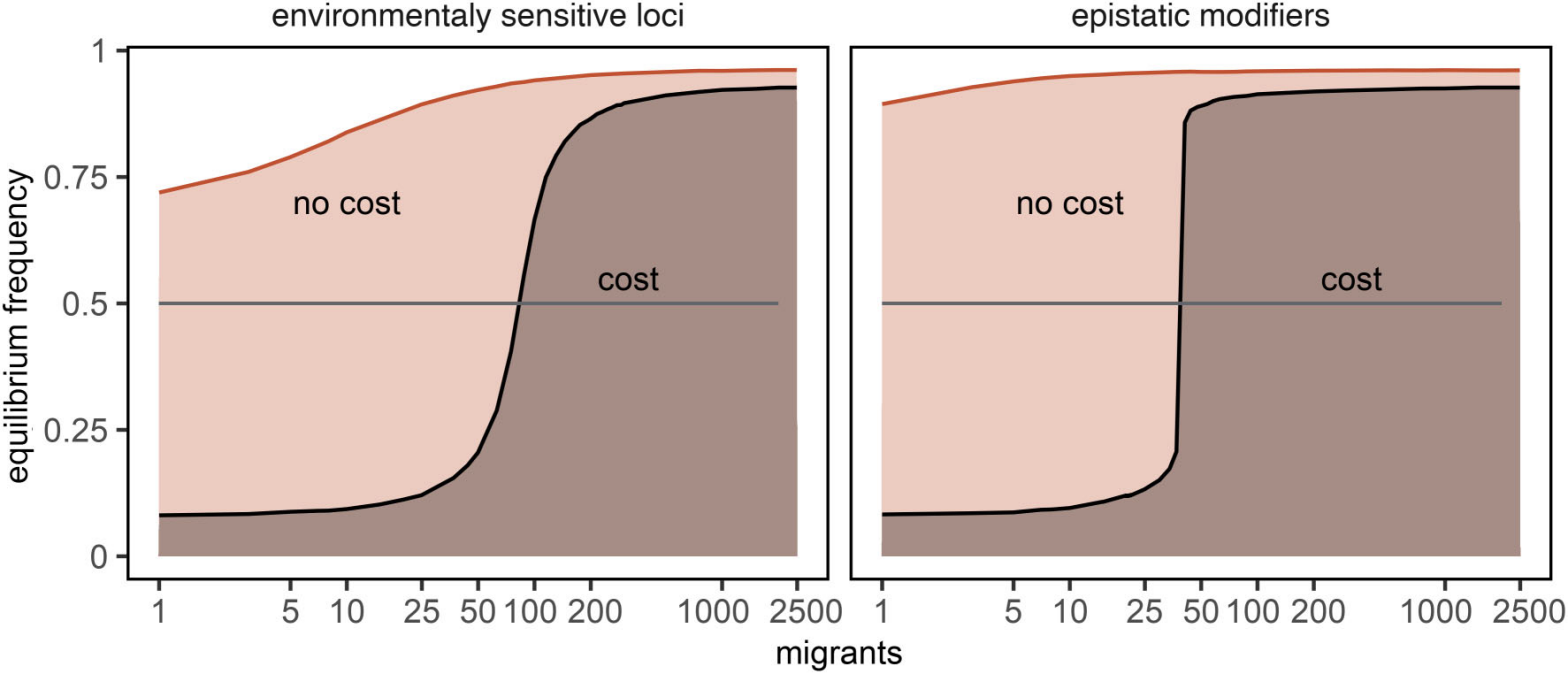
Equilibrium frequencies of plasticity modifier allele (> 0.5 = adaptive plasticity) as a function of migration, recombination, and the cost of plasticity. Forward-in-time evolution (mutation, migration, selection, recombination, and reproduction = sampling) occurred over 100(*N*_A_ + *N*_B_) generations post-burn-in period and 10000 replicates, with curves representing ensemble averages, assuming *N*_A_ = *N*_B_ = 5,000 individuals, *s_j_* = *p_j_* = 0.03, *c_j_* = 0.25*p_j_* or 0, recurrent mutation rate 0.001/*N_j_*, and recombination rate between the modifier and target allele, *r* = 0.0 (environmentally sensitive loci) or 0.5 (epistatic modifiers).

The implementation of recurrent mutation allowed us to compare the evolution of plasticity under two genetic architectures of plastic control and to assess the longevity of plasticity in the face of both recombination between the modifier and target loci and ongoing mutation at both loci, which erode linkage disequilibrium. We find that the adaptive evolution of plasticity under migration holds across different genetic architectures, whether plasticity is governed by environmentally sensitive loci or by interactions between distinct genetic loci (Figure 3). In both models, adaptive plasticity emerges and persists stably over long evolutionary time (100(*N*_A_ *+ N*_B_) generations) across the range of migration rates in the absence of a plasticity cost. Adaptive plasticity requires a non-trivial number of migrants when it incurs a fitness cost.

When comparing the evolution of plasticity under two competing models of plasticity control, interesting patterns emerge—both recombination and reciprocal migration promote plasticity. Under the epistatic model of plasticity control, the adaptive effect of plasticity is marginally stronger at a relatively lower number of migrants. This suggests that free recombination increases the frequency of the plasticity modifier on backgrounds where it confers no benefit. When combined with its advantage on favorable backgrounds, this drives high frequencies of the modifier allele in a population. Nonetheless, the overall effect of recombination remains marginal.

Remarkably, plasticity spreads in the subpopulation of locally adaptive genotypes despite its cost and recurrent mutation, even in the absence of recombination, reaching high frequencies in both populations. Since plasticity confers an advantage to immigrants, from whom it cannot recombine into the residents, the fact that the plasticity allele also spreads within the subpopulation carrying the locally adaptive allele suggests that its population-wide spread is driven by the return of plastic emigrant residents. Therefore, the large equilibrium frequency of plasticity observed in the absence of recombination cannot be explained by unidirectional migration alone, unless immigrants carry plastic alleles due to the evolution of plasticity in the source population. We explore the evolution of plasticity under unidirectional migration in the next section.

### Robustness to model perturbations

The evolution of plasticity is relatively robust to parameter perturbations when the migration is reciprocal and departures from the symmetric model are not extensive. Plasticity that incurs cost becomes unlikely to evolve with increasing asymmetry in selective regimes, migration rates, or population sizes.

**Figure 4.**
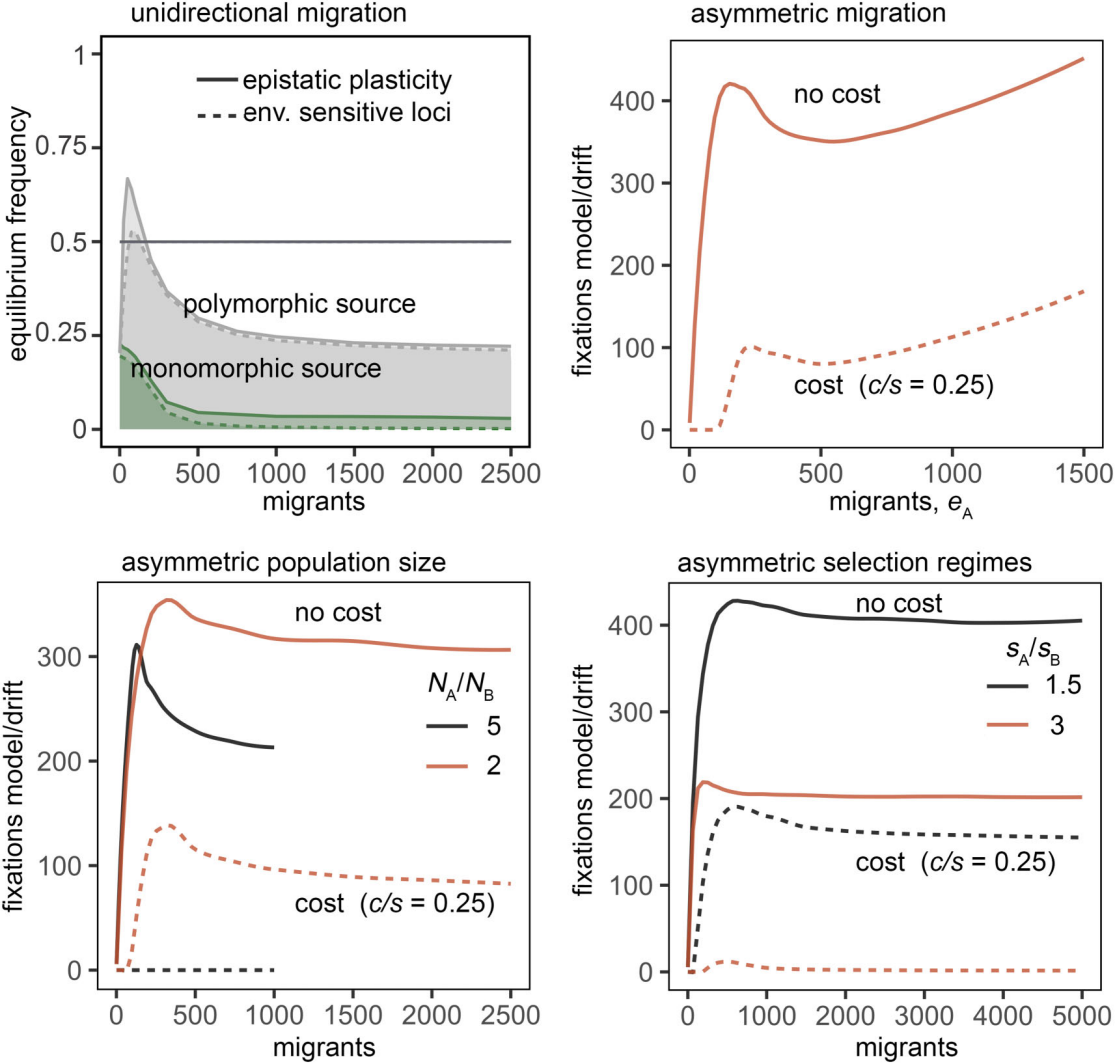
Robustness of the adaptive plasticity to asymmetry in parameters. Top Left: Equilibrium frequency of plasticity modifier allele (> 0.5 = adaptive plasticity) under unidirectional immigration. Simulations were conducted over 100(*N*_A_ + *N*_B_) generations post-burn-in period and 10000 replicates, with curves representing ensemble averages, assuming *N*_A_ = *N*_B_ = 5,000, *s_j_* = *p_j_* = 0.05, *c_j_* = *p_j_*/10 = 0.005, with recurrent mutation in both populations (polymorphic source) or only in receiver population (monomorphic source), with *r* = 0.5 (epistatic plasticity) or *r* = 0 (environmentally sensitive loci). Other panels: Fixation probability of the plasticity modifier allele compared to that under neutrality under single mutant introduction and: (top right) asymmetric migration assuming *e*_B_ = 3*e*_A_, *N_j_* = 10,000, *s_j_* = *p_j_* = 0.03, and *c_j_*/*p_j_* = *c/p* = 0 or 0.25; (bottom left) asymmetric population sizes with *N*_A_ = 10,000 and *N*_B_ = 2,000, *s_i_* = *p_i_* = 0.05 or *N*_B_ = 5,000, *s_j_* = *p_j_* = 0.03, and *c_j_*/*p_j_* = *c*/*p* = 0 or 0.25; and (bottom right) asymmetric selection regimes with *s*_A_ = *p*_A_ = 0.03, *s*_B =_  *p*_B_ = 0.01 or 0.02, and *c_j_*/*p_j_* = *c*/*p* = 0 or 0.25. Simulations were conducted over 100*(N*_A_ + *N*_B_) generations and 1000(*N*_A_ + *N*_B_) replicates, assuming *r* = 0.5.

Can plasticity be adaptive under unidirectional migration? Under a balance between selection and unidirectional migration, we do not observe fixation rates or equilibrium frequencies that would indicate that plasticity is adaptive. Under recurrent mutation at both loci in the population that receives immigrants (with the source population monomorphic for *m*) or in both populations (polymorphic source), even a small cost (e.g., *c_j_* = 0.005 = 0.1*p_j_*) renders plasticity largely maladaptive, especially when the source population does not contribute plasticity alleles (Figure 4, top left). The evolution of adaptive plasticity, thus, depends on its spread across both maladapted and adapted genotypes, enabled by returning emigrants. As migration rates increase and locally adapted individuals move between favorable and harmful habitats, plasticity appears to enhance fitness even among locally adapted genotypes. Therefore, the maintenance of adaptive plasticity requires reciprocal migration, such as in species with high dispersal, where plasticity modifiers are introduced and maintained through multidirectional gene flow among locally adapted populations.

Adaptive plasticity, however, readily evolves under asymmetric bidirectional migration, provided that the asymmetry is not severe, even when costly (Figure 4, top right). Similarly, although asymmetries in population sizes or selection regimes (selection coefficients and the relative costs and benefits of plasticity) reduce the fixation probability of plasticity modifier alleles, plasticity largely remains adaptive. However, when population size asymmetry becomes more pronounced (e.g., 2,000 vs. 10,000), costly plasticity becomes maladaptive (Figure 4, bottom left). Likewise, increasing asymmetry in selection regimes with the proportional costs and benefits of plasticity causes costly plasticity to approach neutrality (e.g., fixation probabilities slightly above 1 but not discernible at the scale shown in Figure 1, bottom right) in parameter ranges where it would otherwise be adaptive under symmetric selection.

To investigate the evolution of plasticity under low migration rates (<1 migrant per generation), we allowed reciprocal migration between two populations only periodically (Figure S3). As migration rates declined, the fixation rates of modifier alleles approached the neutral expectation, indicating only a marginal benefit of plasticity once rates fell below 0.2 migrants per generation (< 2 fixations relative to drift).

### Analytical results

We derive analytical results for symmetric cases using an approximation where the change in genotype frequencies *x_i_* due to selection is modeled as a continuous-time Lotka-Volterra competition model in which the competitive coefficients *α_ik_* are the selection (differential growth rate) on genotype *i* relative to type *k*, ${{\alpha }_{ik}} = {{s}_i} - {{s}_k}$. The selection coefficients *s_i_* are given in Table 1 (Methods) and depend on the population of residence. The selective dynamics in each population are thus given by


\begin{eqnarray*}
\frac{{d{{x}_i}}}{{dt}} = \mathop \sum \limits_k \left( {{{s}_i} - {{s}_k}} \right){{x}_i}{{x}_k}
\end{eqnarray*}

over all genotypes *i, k*. For example, if there are only two genotypes with selective difference *s*, this dynamics results in the familiar logistic growth given by $\frac{{d{{x}_i}}}{{dt}} = s{{x}_i}( {1 - {{x}_i}} )$, whereas for more genotypes, the terms ${{x}_i}(\mathop \sum \limits_k {{\alpha }_{ik}}{{x}_k})$ represent the average selective advantage of type *i* relative to the population mean fitness. In population A, then, the selective dynamics are


\begin{eqnarray*}
\frac{{d{{x}_{am}}}}{{dt}} = {{x}_{am}}\left( {{{c}_A}{{x}_{aM}} + {{s}_A}{{x}_{bm}} + \left( {{{s}_A} + {{c}_A} - {{p}_A}} \right){{x}_{bM}}} \right)
\end{eqnarray*}

\begin{eqnarray*}
\frac{{d{{x}_{aM}}}}{{dt}} = {{x}_{aM}}\left( { - {{c}_A}{{x}_{am}} + \left( {{{s}_A} - {{c}_A}} \right){{x}_{bm}} + \left( {{{s}_A} - {{p}_A}} \right){{x}_{bM}}} \right)
\end{eqnarray*}

\begin{eqnarray*}
\frac{{d{{x}_{bm}}}}{{dt}} = {{x}_{bm}}\left( { - {{s}_A}{{x}_{am}} + \left( {{{c}_a} - {{s}_A}} \right){{x}_{aM}} + \left( {{{c}_a} - {{p}_A}} \right){{x}_{bM}}} \right)
\end{eqnarray*}

\begin{eqnarray*}
\frac{{d{{x}_{bM}}}}{{dt}} = {{x}_{bM}}\left( {\left( { - {{s}_A} - {{c}_A} + {{p}_A}} \right){{x}_{am}} + \left( {{{p}_a} - {{s}_a}} \right){{x}_{aM}} + \left( {{{p}_A} - {{c}_A}} \right){{x}_{bm}}} \right)
\end{eqnarray*}

The selective dynamics in population B have exactly the same formulas with *a*/*b* and A/B indices reversed. Each term ${{\alpha }_{ik}}{{x}_i}{{x}_k}$ in the system of equations is balanced by another term ${{\alpha }_{ki}}{{x}_i}{{x}_k}$. Thus the total frequency is conserved, because ${{\alpha }_{ik}} = \ - {{\alpha }_{ki}}$ and so $\mathop \sum \limits_i \frac{{d{{x}_i}}}{{dt}} = 0$, guaranteeing that $\mathop \sum \limits_i {{x}_i} = 1$ forever.

The constraint on the parameters *s* > *p* > *c* > 0 in each population implies that the selective dynamics in each population have only one stable equilibrium, ${{x}_{am}} = 1$ in population A and ${{x}_{bm}} = 1$ in population B. This is because the growth rate of the non-plastic resident is always positive, while the growth rates of all other genotypes will become negative eventually as the non-plastic resident’s frequency increases. This reflects the intuition that plasticity cannot invade locally adapted populations due to selection alone, because it is not selectively beneficial. To investigate conditions under which plasticity might invade, we must incorporate migration.

Incorporating migration involves adding terms of the form ${{m}_j}( {{{x}_{ij}} - {{x}_{i\tilde{j}}}} )$ to each equation, $\frac{{d{{x}_{ij}}}}{{dt}}$ where here *i* represents genotype and $j\in\{ {{\mathrm{A}},{\mathrm{\ B}}} \}$ encodes which deme is considered. The symbol $\tilde{j}$ means the deme other than deme *j*. These terms homogenize the allele frequencies between the two populations at a rate *m_j_*, where the ratio ${{m}_A}/{{m}_B}$ tunes the relative migrational influence of the populations—how much faster A receives migrants from B. Incorporating migration means that allele frequencies in both demes affect the dynamics, and now eight equations describe the system. One dynamical variable can be eliminated in each deme by noting that $\mathop \sum \limits_i {{x}_i} = 1$, but this still leaves a six-dimensional dynamical system.

The assumption of free recombination and linkage equilibrium between the two loci reduces the system by one further dimension per population. At linkage equilibrium, allele frequencies can be computed from genotype frequencies and vice versa, for example ${{x}_a} = {{x}_{am}} + {{x}_{aM}}$ and ${{x}_{am}} = {{x}_a}( {1 - {{x}_M}} )$. The resulting four-dimensional system is also easier to interpret and write down in terms of deme-specific allele frequencies rather than genotype frequencies:


\begin{eqnarray*}
\frac{{d{{x}_{aA}}}}{{dt}} = {{x}_{aA}}\left( {1 - {{x}_{aA}}} \right)\left( {{{s}_A} - {{p}_A}{{x}_{MA}}} \right) + {{m}_B}\left( {{{x}_{aB}} - {{x}_{aA}}} \right)
\end{eqnarray*}

\begin{eqnarray*}
\frac{{d{{x}_{MA}}}}{{dt}} = {{x}_{MA}}\left( {1 - {{x}_{MA}}} \right)\left( {{{p}_A}\left( {1 - {{x}_{aA}}} \right) - {{c}_A}} \right) + {{m}_B}\left( {{{x}_{MB}} - {{x}_{MA}}} \right)
\end{eqnarray*}

\begin{eqnarray*}
\frac{{d{{x}_{bB}}}}{{dt}} = {{x}_{bB}}\left( {1 - {{x}_{bB}}} \right)\left( {{{s}_B} - {{p}_B}{{x}_{MB}}} \right) + {{m}_A}\left( {{{x}_{bA}} - {{x}_{bB}}} \right)
\end{eqnarray*}

\begin{eqnarray*}
\frac{{d{{x}_{MB}}}}{{dt}} = {{x}_{MB}}\left( {1 - {{x}_{MB}}} \right)\left( {{{p}_B}\left( {1 - {{x}_{bB}}} \right) - {{c}_B}} \right) + {{m}_A}\left( {{{x}_{MA}} - {{x}_{MB}}} \right)
\end{eqnarray*}

Here ${{x}_{ij}}$ refers to the frequency of the allele *i* at its respective locus, marginalized over the other locus, in deme *j*. Each equation pits selection against migration in two terms: the selective term is of the form $x( {1 - x} )s$, where *s* in turn depends on the allele frequency at the other locus, whereas the migration term $m( {x - \tilde{x}} )$ depends on the frequency of the same allele in the other population.

Selection for non-plastic adaptation to the local deme under these assumptions is always positive, since by definition $s > p$. Selection for plasticity, on the other hand, is positive if and only if


\begin{eqnarray*}
x < 1 - \frac{c}{p}
\end{eqnarray*}

where *x* is the frequency of the resident allele. For example, if migration-selection balance holds the resident allele frequency at 0.9, plasticity is positively selected only if the cost is less than 0.1 times the benefit. The sign of selection for plasticity depends only on $p/c$ and in particular does not depend on *s* or the ratio of *s* and *p*—but does depend on the frequency of resident versus immigrant alleles at the target locus. The relative magnitudes of *m, s*, and *p* then determine the equilibrium frequency of the target allele, which in turn also depends on the frequency of the plasticity allele.

To gain intuition about this complicated feedback process, we investigate a particular symmetric case in which the parameters ${{s}_A} = {{s}_B} = s$, ${{p}_A} = {{p}_B} = p$, etc. are the same in both populations and the initial conditions are mirrored. That is, given the initial condition ${{x}_{aA}} = {{x}_{bB}} = {{x}_0}$, ${{x}_{MA}} = {{x}_{MB}} = {{x}_{M0}}$, the dynamics $\frac{d}{{dt}}( {{{x}_{aA}} - {{x}_{bB}}} )$ and $\frac{d}{{dt}}( {{{x}_{MA}} - {{x}_{MB}}} )$ are initially equal to 0, and therefore remain zero for all time. This means that if two populations with equal parameters are initialized with equal frequencies of the plasticity allele and opposite frequencies of the target allele$,\ {{x}_{aA}} = 1 - {{x}_{aB}}$, then the trajectories deterministically evolve so that the plasticity allele frequencies remain the same in both populations and the target allele frequencies remain opposite. In this setting, we need only track the trajectories in a single population, and the deterministic system becomes


\begin{eqnarray*}
\frac{{dx}}{{dt}} = x\left( {1 - x} \right)\left( {s - p{{x}_M}} \right) + m\left( {1 - 2x} \right)
\end{eqnarray*}

\begin{eqnarray*}
\frac{{d{{x}_M}}}{{dt}} = {{x}_M}\left( {1 - {{x}_M}} \right)\left( {p\left( {1 - x} \right) - c} \right)
\end{eqnarray*}

for the target resident allele frequency *x* and plasticity allele frequency ${{x}_M}$ in both populations.

In this simple case, the plasticity allele is either fixed or lost in both populations, depending on the parameters and sometimes the initial conditions. The system behavior is determined by the nullclines, as shown in Figure S4. The allele frequency ${{x}_M}$ is stationary on the nullcline $\frac{{d{{x}_M}}}{{dt}} = 0$, that is when $x = 1 - \frac{c}{p}$, independent of ${{x}_M}$. The *x* nullcline $\frac{{dx}}{{dt}} = 0$ is easier to write in terms of ${{x}_M}.$ That is, as a function of a given ${{x}_M}$, the nullcline is the migration-selection equilibrium given by


\begin{eqnarray*}
{{x}^*}\left( {{{x}_M}} \right) = \frac{1}{2} - \frac{m}{{s - p{{x}_M}}} + \sqrt {{{{\left( {\frac{1}{2}} \right)}}^2} + {{{\left( {\frac{m}{{s - p{{x}_M}}}} \right)}}^2}}.
\end{eqnarray*}

This expression is bounded by 1 when $\frac{m}{{s - p{{x}_M}}}$ goes to zero and $\frac{1}{2}$ when $\frac{m}{{s - p{{x}_M}}}$ goes to infinity, matching intuition that zero migration causes local adaptation to fix and strong migration homogenizes allele frequencies to $\frac{1}{2}$. With plasticity fixed, the equilibrium depends on $\frac{m}{{s - p}}$ whereas the equilibrium depends on $\frac{m}{s}$ when plasticity is absent. Because $\frac{m}{{s - p}} > \frac{m}{s}$ in general, ${{x}^*}( 1 ) < {{x}^*}( 0 )$. That is, plasticity depresses ${{x}^*}$ towards $\frac{1}{2}$, consistent with the idea that plasticity masks the effects of selection and thus shifts the migration-selection balance closer to homogenization.

Three outcomes are possible depending on the parameters. If ${{x}^*}( 1 ) > 1 - \frac{c}{p}$, then the *x* nullcline lies entirely to the left of the ${{x}_M}$ nullcline, and plasticity eventually goes extinct from any initial condition (Figure S4 right, bottom panels). Intuitively, this is because plasticity is negatively selected even when it is fixed. If ${{x}^*}( 0 ) < 1 - \frac{c}{p}$, the opposite situation occurs, the *x* nullcline lies entirely to the left of the ${{x}_M}$ nullcline and plasticity fixes because it is positively selected under conditions of mutation-selection equilibrium even when it is rare (Figure S4 right, top panels). Hysteresis arises when ${{x}^*}( 1 ) < 1 - \frac{c}{p} < {{x}^*}( 0 )$. In this case, the nullclines intersect at an unstable interior equilibrium, and the system exhibits bistability for the fixation or loss of plasticity (Figure S4, left). If plasticity is initially common, the migration-selection equilibrium is suppressed to the point that plasticity is still positively selected. If plasticity is lost, however, it is no longer positively selected under the new migration-selection equilibrium, and new plasticity mutants cannot invade. Changing conditions, such as increased migration, might lead to the fixation of plasticity. Once fixed, plasticity suppresses the migration-selection equilibrium, facilitating its own conditions of success. Following fixation, an even lower migration rate is required before plasticity is negatively selected again.

The condition for new plasticity mutants to be favored by selection is therefore


\begin{eqnarray*}
\frac{c}{p} < \frac{1}{2} + \frac{m}{s} - \sqrt {{{{\left( {\frac{1}{2}} \right)}}^2} + {{{\left( {\frac{m}{s}} \right)}}^2}}.
\end{eqnarray*}

This expression can be interpreted as the cost of plasticity relative to the benefit must be less than the probability of “needing” plasticity due to local maladaptation. That probability is, in turn, determined fully by the ratio of migration to selection strengths. For very low migration rates, the right-hand side reduces to approximately *m*/*s*, while for high migration, the right-hand side asymptotes to 1/2. Thus, a necessary condition for plasticity to evolve is *c*/*p* < *m*/*s*.

## Discussion

Evolution of phenotypic plasticity is typically envisioned to occur after environmental change renders non-plastic genotypes maladaptive. Here, we theorize that plasticity can be selected for in locally adapted populations in constant environments due to migration, even when plasticity is costly and not associated with the fittest genotype, irrespective of the underlying genetic architecture. This evolution of plasticity without changing abiotic conditions highlights migration as an underappreciated mechanism that may contribute to the prevalence of plasticity in nature. More broadly, this role of migration invites a rethinking of what constitutes an “environment”: Gene flow between ecologically distinct habitats can function as an adverse pressure across generations, potentially maintaining costly traits.

Migration is ubiquitous in nature. Distinct populations exchange genes through many mechanisms: birds through natal dispersal and seasonal movements (Heikkinen et al., 2020); mammals via long-distance travel (Thompson & Jenks, 2010); marine organisms through larval drift (Bett et al., 2017); insects by flight or passive transport (Chapman et al., 2015); and plants through pollen and seed dispersal (Browne et al., 2018; Nathan, 2006). Microbes migrate via air, water, or host vectors (Pearman et al., 2024). In organisms with generation times that exceed the scale of environmental variation, which can buffer the effects of temporally varying selection (Gamelon et al., 2014; Le Coeur et al., 2022), migration may serve as a key driver of plasticity. Scenarios similar to those studied here arise under bidirectional partial migration, where populations consist of both migrants and residents. Such migration patterns are widespread across long-lived taxa, including birds (Lundberg, 1988) and ungulates (Berg et al., 2019). At a global scale, trade and transport can also facilitate large-scale exchanges: shipping routes repeatedly move large groups of organisms via ballast water, creating multidirectional propagule flow across ports (Briski et al., 2013; Seebens et al., 2013; Sardain et al., 2019).

Yet previous studies of migration between distinct populations have only predicted evolution favoring plasticity under source-sink dynamics (Promy et al., 2023), in combination with temporally varying selection (Carja et al., 2014), or within deterministic models assuming standing variation in plasticity (Sultan and Spencer, 2002; Chevin & Lande, 2011). Sultan and Spencer 2002) found that migration broadens the conditions for the evolution of plasticity, both with and without temporal variation, when specialists and a plastic type are present in a metapopulation, in the absence of recombination or genetic drift. In the scenarios investigated so far, however, models assumed either the absence of local adaptation in one population, temporally varying selection across generations, or pre-existing plasticity. In contrast, our study reveals that gene flow can favor the emergence of phenotypic plasticity in locally adapted finite populations, without requiring varying environments or pre-existing standing genetic variation. The outsized influence of migration also suggests that identifying patterns of gene flow among locally adapted populations may help predict rapid plastic response in the face of future environmental changes in habitat or identify species likely to become invasive (Lee et al., 2003; Richards et al., 2006).

That plasticity will be selectively favored despite competing with locally fitter genotypes in a population may run counter to intuition. Yet, in our model, plasticity emerges as an adaptive trait, although it is not associated with the fittest genotype. How is this possible? The evolution of plasticity is driven by immigration, including both returning plastic emigrants and non-local individuals. Rather than merely shifting the migration–selection balance, reciprocal migration transforms plasticity from maladaptive to adaptive once the migration rate, relative to the strength of selection, exceeds the cost-to-benefit ratio of plasticity (*m*/*s* > *c*/*p*). Moreover, our analytical results point to hysteresis, where plasticity can be maintained by selection under a wider range of conditions than are required for it to invade. The observation that selection can promote plasticity even when plastic phenotypes are less fit than non-plastic residents helps explain the emergence of seemingly maladaptive plasticity (Cenzer, 2017).

Our results highlight that migration modulates the nature of selective environments. Reciprocal migration may serve as an environmental disturbance that reshapes the fitness landscape, potentially turning maladaptive traits, like costly plasticity, into adaptive ones, despite competing locally-adapted genotypes. By extension, low and potentially undetectable rates of migration may explain the persistence of other costly traits, such as energetically expensive sensory systems or genome size in microbes.

Our study demonstrates the emergence of plasticity due to migration in an analytically tractable model. We assume constant fitnesses and population sizes—common assumptions and useful simplifications in population genetics. When plasticity-conferring alleles arise and fix consecutively without interference, this model extends to polygenic plasticity (Ewens, 2004; Gillespie, 2004). However, by assuming constant population size, we model soft selection, where the fitness of an allele is defined relative to competing genotypes (Wallace, 1975). Here, plastic immigrants increase in frequency by capitalizing on selection against maladapted non-plastic immigrants. In nature, however, population sizes fluctuate, relaxing the strength of density dependence. Evaluating the evolution of plasticity under the interplay of soft and hard selection (no density dependence), as well as the effects of changing density on migration, requires eco-evolutionary models that incorporate demographic fluctuations (e.g., Rowland & Gulisija, 2025). The complexity of nature also involves fluctuating migration and fitness landscapes. Further analysis of complex and realistic scenarios is required to explore the plasticity-promoting effect of migration under a broader and more realistic range of conditions.

## Supplementary Material

qraf040_Supplemental_File

## Data Availability

The code used to generate the results is publicly available on Dryad, https://doi.org/10.5061/dryad.37pvmcvzg.
