# Supplementary material for: Evolution of phenotypic plasticity owing to migration
Source: Evol Lett. 2025 Nov 8;10(1):31–41. doi: 10.1093/evlett/qraf040 (PMC12870872; doi:10.1093/evlett/qraf040)
Supplement: qraf040_Supplemental_File [file qraf040_supplemental_file.pdf]

Evolution of phenotypic plasticity owing to migration

Supplementary Materials

Davorka Gulisija[1,2*], and

Mitchell Newberry[1]

[1] Department of Biology, University of New Mexico, USA

[2] Department of Computer Science, University of New Mexico, USA

[*]Correspondence: dgulisija@unm.edu

Davorka Gulisija

219 Yale Boulevard NE

3566 Castetter Hall

Albuquerque, NM 87131

United States

Supplementary Figures

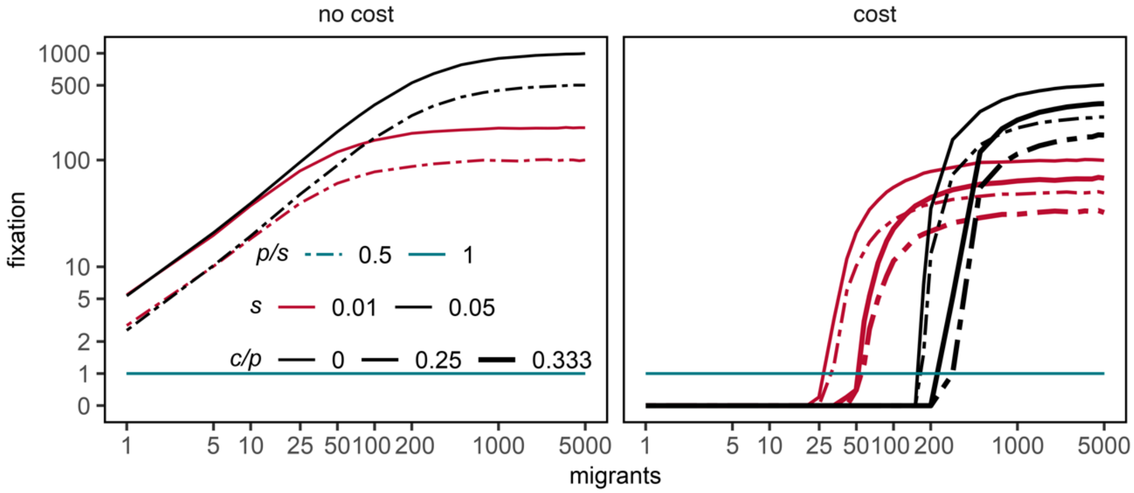

Figure S1. **Emergence of plasticity owing to migration.** Fixation probability of a plasticity modifier allele relative to that under drift (>1 indicates adaptive plasticity; horizontal green line = neutrality) as a function of the number of migrants between populations (given on a log scale), selection coefficient, plasticity benefit, and the cost of plasticity. Simulations assumed a single plasticity modifier allele introduction to a monomorphic non-plastic meta-population and forward-in-time evolution (migration, selection, recombination, and reproduction = sampling) until the modifier allele is fixed or lost over $1000(N_A + N_B)$ replicate runs, assuming $N_A = N_B = 10{,}000$ individuals and free recombination between the plasticity and target locus (epistatic plasticity).

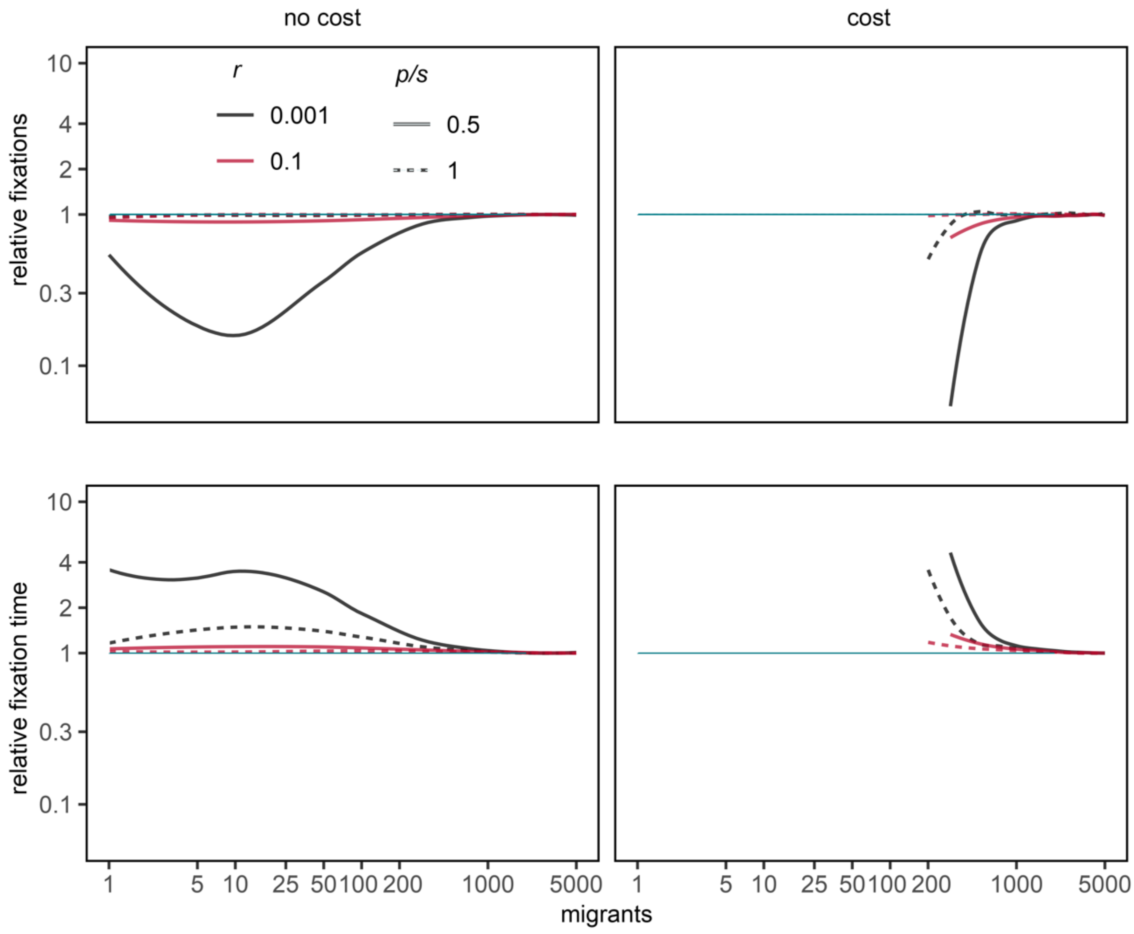

Figure S2. **Evolution of a linked plasticity modifier allele.** The plots show the fixation probability and fixation time of a plasticity modifier allele relative to that under free recombination (values >1 indicate a higher probability of fixation or longer fixation time; values <1 indicate the opposite) as a function of the number of migrants between populations per generation (given on a log scale), plasticity benefit, *p*, and the cost of plasticity, for $c_j = 0.33p_j$. A horizontal green line marks the expectation under free recombination ($r = 0.5$). Note that although linkage reduces fixation efficiency, the plasticity modifier remains adaptive—fixation probabilities still exceed neutrality as under free recombination. Simulations were run with a single introduction of a plasticity modifier allele into a monomorphic, non-plastic metapopulation. Forward-in-time evolution was simulated until the modifier allele is fixed or lost over $1000(N_A + N_B)$ replicate runs, assuming $N_A = N_B = 10{,}000$ individuals and $s_A = s_B = 0.03$.

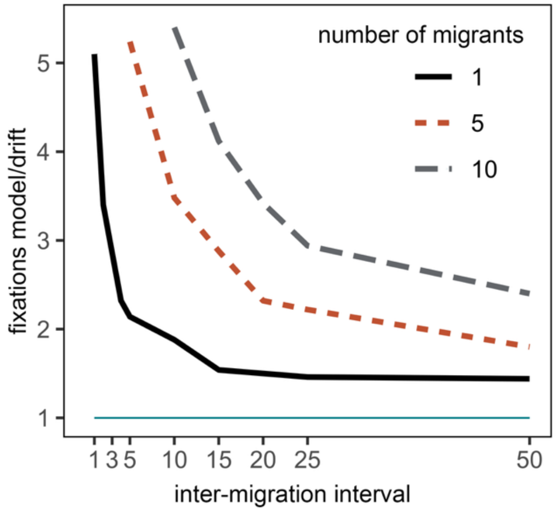

Figure S3. **Evolution of plasticity under low-rate migration.** Fixation probability of a plasticity-modifier allele relative to neutral expectation (>1 indicates adaptive plasticity; horizontal green line = neutrality) plotted as a function of the interval between migration events (1, 2, 3, 4, 5, 10, 15, 20, 25, 50, or 100 generations) and the number of migrants per event (1, 5, or 10). Simulations introduce a single plasticity modifier into a monomorphic, non-plastic metapopulation; the modifier confers a fitness benefit $p_i = s_i = 0.03$, and no cost. Forward-in-time evolution (migration, selection, recombination, and reproduction = sampling) was conducted until the modifier allele is fixed or lost over $1000(N_A + N_B)$ replicate runs, assuming $N_A = N_B =$ 10,000 individuals and free recombination between the plasticity and target locus (epistatic plasticity). The height of the lines corresponds to relative fixation probabilities as a function of the inter-migration interval, with expected number of migrants per generation = (number of migrants)/(interval between migration events). The leftmost point on each line corresponds to one migrant per generation.

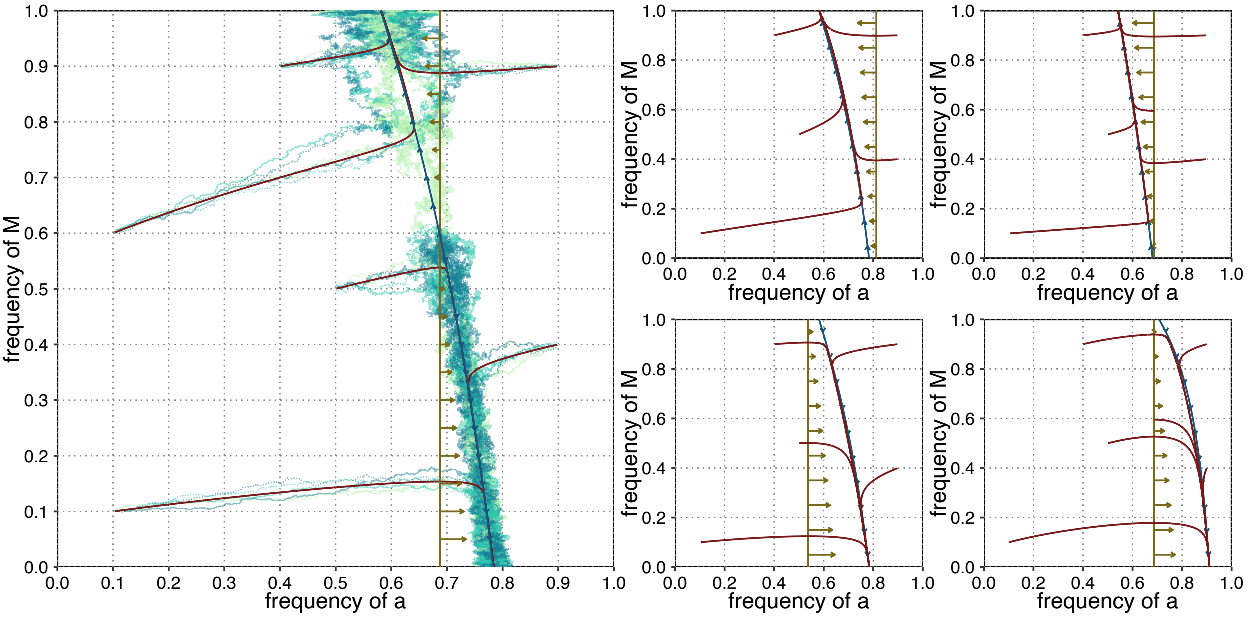

Figure S4. Allele frequency phase portraits across parameter space. Left: Fixation of plasticity (x_M) exhibits bistability and hysteresis. Trajectories (red: deterministic, blue-green: stochastic) rapidly approach the vicinity of migration-selection balance (blue nullcline), then slowly fix or lose plasticity. Right, first column: modulating c/p displaces the $dx_M/dt = 0$ nullcline (yellow) between regimes where plasticity always fixes (top) or always goes extinct (bottom). Last column: modulating m/s displaces the $dx_a/dt = 0$ nullcline (blue) between regimes where plasticity always fixes (top) or always goes extinct (bottom).
